# BioHPP in prosthetic dentistry: a narrative review of mechanical, biological, and clinical properties

**DOI:** 10.2340/biid.v13.45303

**Published:** 2026-01-02

**Authors:** Aslı Bengisu Karayel Gerçek, Ragibe Şenay Canay

**Affiliations:** Department of Prosthodontics, Faculty of Dentistry, Hacettepe University, Ankara, Türkiye

**Keywords:** BioHPP, polyetheretherketone, high-performance polymer, prosthetic dentistry, dental implants, adhesion, CAD/CAM, biocompatibility, mechanical properties, prosthodontics

## Abstract

Biocompatible high-performance polymer (BioHPP), a high-performance polymer derived from polyetheretherketone (PEEK) and reinforced with ceramic fillers, has emerged as a promising alternative to conventional metal and ceramic framework materials in prosthetic dentistry. With an elastic modulus (~4 GPa) comparable to that of cortical bone, BioHPP promotes physiological stress distribution and reduces stress shielding, thereby supporting peri-implant bone preservation. Its excellent chemical inertness and low bacterial affinity minimize mucosal inflammation and reduce the risk of peri-implant disease. However, the material’s low surface energy and hydrophobicity pose challenges to long-term adhesive stability, necessitating specific surface modification techniques and specialized adhesive systems. Clinically, BioHPP has been successfully applied in single crowns, fixed partial dentures, full-arch hybrid prostheses (e.g. All-on-Four), bar-retained overdentures, maxillofacial frameworks, customized abutments, and provisional restorations. Despite its favorable biomechanical and biological profile, limitations such as inherent opacity, restricted fracture toughness, and the scarcity of long-term clinical data highlight the need for further interdisciplinary research and material innovation. This narrative review comprehensively evaluates the mechanical, biological, and adhesive characteristics of BioHPP, compares its performance with traditional framework materials, and discusses its clinical applications and future perspectives in prosthetic rehabilitation.

## Introduction

In recent years, the demand for metal-free restorative materials in prosthetic dentistry has increased significantly due to the growing emphasis on biocompatibility, esthetic excellence, and optimal functional performance. The limitations associated with metal-based substructures, hypersensitivity reactions, ion release, mucosal discoloration, and suboptimal esthetic outcomes, have prompted a paradigm shift toward high-performance polymer-based alternatives [1]. Moreover, metallic components frequently produce radiographic artifacts in advanced imaging modalities such as cone-beam computed tomography (CBCT), thereby compromising diagnostic precision and disrupting digitally driven treatment workflows [2].

Amid these advancements, polyetheretherketone (PEEK) and its ceramic-reinforced derivative, BioHPP (biocompatible high-performance polymer), have garnered increasing interest as advanced framework materials for diverse prosthodontic applications [3]. With an elastic modulus of approximately 3–4 GPa, which closely resembles that of cortical bone, BioHPP promotes favorable stress distribution around implant-supported restorations. Additional advantages of BioHPP include its low density, exceptional chemical inertness with negligible ion release, minimal plaque accumulation, and complete compatibility with modern Computer-Aided Design / Computer-Aided Manufacturing (CAD/CAM) workflows [4–6].

BioHPP has demonstrated clinical versatility across a wide range of prosthetic applications, including single-unit crowns, multi-unit fixed dental prostheses, All-on-Four hybrid restorations, bar-retained implant overdentures, maxillofacial prosthetic frameworks, customized abutments, and provisional components. The shift to high-performance polymers is mainly driven by their superior biological compatibility and improved esthetic integration, especially in patients with metal hypersensitivity or clinical situations requiring optimal peri-implant soft tissue stability [1, 7].

This narrative review aims to critically examine the structural characteristics, clinical applications, and biomechanical behavior of BioHPP within the context of prosthetic dentistry. In addition, it highlights current evidence gaps and proposes future research directions to enhance the clinical integration and long-term success of high-performance polymer-based restorations.

## Material background and composition

High-performance polymers from the polyaryletherketone (PAEK) family, particularly PEEK and its derivatives, have garnered increasing interest in prosthetic dentistry due to their favorable biomechanical behavior and exceptional chemical stability [1, 8]. Members of the PAEK family, including polyetherketone (PEK), PEEK, and polyetherketoneketone (PEKK), exhibit distinct molecular architectures that influence their thermal stability, degree of crystallinity, and compatibility with CAD/CAM processing technologies [9, 10]. PEEK has gained widespread clinical acceptance due to its superior mechanical properties, chemical stability, and well-documented biocompatibility profile [11]. Moussa et al. [2] clinically validated the effectiveness of PEEK-based BioHPP^®^ bars, highlighting their potential to promote better soft tissue health and higher patient satisfaction compared to traditional Co-Cr bars.

BioHPP, a PEEK-based composite reinforced with 20% ceramic fillers, represents a significant advancement in this category. The integration of submicron ceramic particles enhances stiffness, raising the elastic modulus to approximately 3–4 GPa, which better matches cortical bone and facilitates favorable stress transfer in implant-supported prostheses. Additionally, these reinforcements improve surface hardness, wear resistance, and long-term dimensional stability [6, 8].

In contrast to sintered or cast conventional framework materials, BioHPP is amenable to efficient subtractive processing via computer-aided manufacturing (CAM), facilitating seamless integration into digital workflows while reducing laboratory time and promoting precision, consistency, and clinical reproducibility [1, 12].

Given these advantages, BioHPP has progressed beyond fixed prosthetic frameworks and is increasingly utilized in various clinical contexts, including bar-supported removable prostheses, provisional restorations, customized abutments, and maxillofacial prosthetics [2, 3].

## Mechanical properties and biomechanical behavior

### Elastic modulus and stress distribution

One of the defining mechanical attributes of BioHPP in prosthetic dentistry is its elastic modulus, which closely approximates that of human cortical bone. With values ranging from 3 to 4 GPa, BioHPP exhibits significantly lower stiffness compared to traditional framework materials such as titanium (~110 GPa) and zirconia (~200 GPa) [1, 13]. This mechanical compatibility is vital in reducing stress shielding effects, facilitating a more physiological transmission of occlusal loads to the peri-implant bone [8, 14].

In an in vitro study, Elshamy et al. [15] demonstrated that implant-supported fixed partial dentures fabricated with BioHPP frameworks generated significantly lower peri-implant microstrain compared to those with metal frameworks, while showing no statistically significant difference compared to zirconia frameworks. Although the differences between BioHPP and zirconia were not statistically significant, the stress distribution pattern associated with BioHPP was notably more favorable.

These findings highlight the material’s potential utility in full-arch rehabilitations, posterior segment prostheses, and cantilevered designs where optimized load transfer is essential. As noted by Andrikopoulou et al. [16], the material’s viscoelastic behavior plays a key role in dissipating functional masticatory forces, thereby minimizing the risk of debonding and enhancing the mechanical stability of resin-bonded fixed dental prostheses. This damping capacity boosts overall prosthesis retention and contributes to patient comfort.

Additionally, incorporating ceramic fillers enhances BioHPP’s fracture toughness and resistance to plastic deformation [2, 17]. These properties make it particularly advantageous in high-stress clinical scenarios. Importantly, BioHPP’s low modulus promotes bone preservation and replicates the biomechanical response of natural teeth, supporting better functional integration and long-term success [1, 18].

### Fatigue and fracture resistance

In addition to its bone-mimetic elastic modulus, BioHPP demonstrates outstanding fatigue resistance under cyclic loading conditions – a critical factor for ensuring long-term prosthetic durability. In vitro fatigue simulations have revealed that BioHPP frameworks can consistently withstand fracture loads exceeding 1200 N, which surpasses the average occlusal forces observed in the posterior dentition [3, 19]. These results emphasize BioHPP’s mechanical reliability, particularly in prosthetic designs subjected to high occlusal loads. According to Elsebai et al. [20] CAD/CAM-fabricated BioHPP dentures exhibited slightly higher fracture resistance than zirconia-reinforced alternatives, although both materials significantly improved the prosthesis’ mechanical performance compared to non-reinforced dentures.

Including ceramic microfillers in BioHPP enhances stiffness and significantly improves its ability to absorb and dissipate energy during functional loading. This characteristic reduces mechanical complications, especially in implant-supported fixed partial dentures and bar-retained removable prostheses [1, 2, 21].

By combining high fracture toughness with effective energy damping, BioHPP offers both structural durability and functional resilience. These features render it a robust and reliable material for complex, load-bearing prosthetic rehabilitations where long-term performance is crucial.

## Biological properties and biocompatibility

### Tissue compatibility and cellular response

BioHPP, a high-performance polymer derived from PEEK, retains the chemical inertness and well-established biocompatibility of its base matrix, making it highly suitable for intraoral use. The lack of metal ion release and reactive degradation products significantly decreases the risk of cytotoxicity, hypersensitivity, or adverse inflammatory responses in both soft and hard tissues [8, 22]. In vitro studies utilizing osteoblast and fibroblast cultures have shown that BioHPP exhibits no cytotoxic or mutagenic potential. Furthermore, when modified with an appropriate surface topography, BioHPP has been shown to support cellular adhesion and proliferation, key processes for successful peri-implant soft and hard tissue integration [3, 23].

Due to its biological inertness, BioHPP elicits minimal inflammatory and immunological responses, thereby contributing to the maintenance of peri-implant soft tissue health. In contrast to metallic materials such as titanium or cobalt–chromium alloys, BioHPP does not provoke mucosal pigmentation and maintains its optical properties over time (2). According to histological evaluations by Lo Giudice et al. [24], BioHPP restorations are associated with reduced inflammatory cell infiltration and the establishment of a stable epithelial–connective tissue interface around BioHPP-based restorations, which is consistent with Discepoli et al.’s [25] findings indicating minimal inflammatory response and similar soft tissue healing around PEEK and titanium abutments.

From a biomechanical perspective, BioHPP’s elastic modulus of approximately 4 GPa closely resembles that of cortical bone, allowing for the effective transmission of functional forces to the surrounding bone. This characteristic reduces stress shielding, preserving peri-implant bone architecture and enhancing osseointegration [21, 26]. Furthermore, its viscoelastic properties provide mechanical buffering in high-load environments, thereby improving long-term functional performance [1, 2].

### Biofilm formation and peri-implant soft tissue behavior

The surface characteristics of BioHPP – characterized by low surface energy and intrinsic hydrophobicity – provide both clinical and microbiological benefits. These physicochemical attributes enhance resistance to hydrolytic degradation and thermal aging, while also reducing microbial adhesion, compared to conventional metallic frameworks such as titanium and cobalt–chromium alloys. Almuhayya et al. [27] demonstrated in their in vitro study that polished or hydrophilically modified BioHPP surfaces significantly diminish biofilm formation and microbial colonization. This reduced biofilm accumulation is particularly beneficial in prosthetic designs with extensive surface areas, such as full-arch fixed prostheses and bar-retained overdentures, where effective plaque control and maintenance of peri-implant soft tissue integrity are critical for long-term clinical success. Although preliminary clinical evidence indicates a reduced incidence of peri-implantitis in BioHPP-based restorations, these observations warrant further validation through well-designed, long-term randomized clinical trials [1, 2].

According to Moussa and El Afandy [2] BioHPP bars used in mandibular implant-retained overdentures exhibit more favorable biological behavior in peri-implant soft tissues compared to cobalt–chromium frameworks. The authors reported significantly reduced vertical bone loss and improved peri-implant mucosal health over 12 months, suggesting a superior soft tissue response associated with BioHPP infrastructure. These favorable clinical outcomes stem from BioHPP’s intrinsic biocompatibility and its bone-matching elastic modulus, which facilitates the transmission of physiological masticatory forces and promotes peri-implant structural stability. In addition, its smooth, low-retentive surface reduces microbial adherence, preventing peri-implant mucositis [8, 28].

These findings underscore the potential of BioHPP as a next-generation substructure material that promotes peri-implant tissue health by minimizing bacterial biofilm formation, improving soft tissue integration, and mitigating chronic inflammatory responses.

## Adhesion and bonding characteristics

### Surface modification techniques

Despite its favorable mechanical and biological attributes, BioHPP presents notable challenges in adhesive dentistry due to its intrinsically low surface energy and hydrophobic molecular molecular structure. Unlike silane-based coupling agents that are effective with silica-containing ceramics or oxide-mediated bonding strategies suitable for metals, conventional adhesive methods are inadequate for achieving durable chemical adhesion to BioHPP’s non-polar and chemically inert surface. Consequently, advanced surface modification techniques aimed at enhancing wettability and micromechanical retention are essential for optimizing bonding performance in restorative applications [29].

Among the most widely used physical surface treatments is airborne-particle abrasion with aluminum oxide (Al₂O₃) particles, which range from 50 to 110 µm. This method increases surface roughness, facilitating micromechanical interlocking with veneering composite resins. Combined with oxygen plasma treatment, it can further increase surface energy, resulting in significantly enhanced shear bond strength [29].

In addition to physical methods, chemical surface modification strategies have shown promising results. According to recent findings by Luo et al. [29] using sulfuric acid etching with 98% H₂SO₄ for 60 seconds to effectively introduce sulfonic acid functional groups onto the BioHPP surface, thereby enhancing its chemical reactivity and significantly improving the adhesive interface with resin composites, resulting in shear bond strength values exceeding 27 MPa. Furthermore, low-pressure plasma activation using hydrogen–oxygen gas mixtures has been reported to elevate surface energy and microroughness simultaneously. These modifications enhance adhesion to resin-based systems and promote improved cellular interactions, thereby reinforcing BioHPP’s functional and biological performance [29].

### Adhesive systems and clinical implications

The low surface energy and hydrophobic nature of BioHPP limit the formation of strong chemical and micromechanical bonds with restorative materials. To address this challenge, bonding systems incorporating functional monomers with phosphate groups, particularly 10-methacryloyloxydecyl dihydrogen phosphate (10-MDP), and methyl methacrylate (MMA)-based agents have been developed. When applied following optimized surface conditioning protocols, these monomers form durable chemical interactions with the modified BioHPP surface, thereby improving bond strength and overall restoration integrity [30, 31].

Primer systems engineered explicitly for PAEK-based polymers have been shown to enhance monomer infiltration following surface activation, thereby facilitating durable adhesive bonding [6]. Nevertheless, concerns persist regarding the long-term stability of such adhesive interfaces. Gabra et al.[32] reported a significant reduction in bond strength after thermal aging, notably when primer application was omitted from the bonding protocol. Conversely, El-Wassefy [33] documented improved bond strength following thermocycling under optimized surface treatment conditions. The inconsistency between these findings, along with the lack of cyclic mechanical loading evaluation in the study by Gonçalves et al. [34], underscore the critical need for comprehensive, evidence-based protocols that ensure the long-term adhesive performance of PAEK-based restorative systems under functional loading conditions.

Achieving optimal bonding outcomes in BioHPP restorations requires careful consideration of both the adhesive system and the surface preparation sequence. In clinical procedures such as the cementation of fixed prostheses or composite veneering for esthetic purposes, a combined approach involving mechanical roughening, plasma treatment, and the application of BioHPP-compatible primers is recommended to ensure reliable adhesion [35].

Despite these advancements, a universally accepted bonding protocol has yet to be established. This underscores the need for ongoing development of surface-specific primers and adhesive systems designed to match the physicochemical characteristics of BioHPP, particularly to ensure consistent clinical performance across a range of restorative scenarios.

## Clinical applications of BioHPP

### Fixed implant-supported prostheses

BioHPP has gained increasing clinical attention as an alternative to conventional zirconia and metal alloy frameworks in fixed implant-supported prostheses, particularly in full-arch rehabilitations and hybrid prosthetic designs. Its favorable biomechanical profile and improved clinical handling support its integration into contemporary prosthodontic workflows. These frameworks may be veneered with composite resins or ceramics through adhesive cementation protocols, offering esthetic flexibility and efficient intraoral repair options [35, 36].

BioHPP’s significantly lower weight compared to metal alloys enhances patient comfort. Furthermore, its radiolucency removes imaging artifacts in radiographic evaluations, enabling more accurate long-term clinical monitoring [37].

Due to its exceptional fracture and cyclic fatigue resistance, BioHPP is a dependable framework material in full-arch implant-supported rehabilitations. Its intrinsic shock-absorbing capacity and biomimetic stress modulation properties contribute to the protection of osseointegrated implants, enhancing masticatory function. According to the findings of Aboelnagga et al. [38] and Shash et al. [39] high-performance polymer frameworks, such as BioHPP, PEKK, and PEEK, exhibit favorable stress and strain distribution patterns, making them especially suitable for load-intensive applications, including the All-on-Four prosthetic rehabilitation approach. Moreover, the material’s compatibility with CAD/CAM systems enables the precise fabrication of patient-specific restorations, improving procedural efficiency and reproducibility [12].

Nevertheless, certain secondary mechanical complications, particularly the chipping of veneering materials, have been reported in clinical settings. Although such issues are often manageable without necessitating full prosthesis replacement, they underscore the need for comprehensive longitudinal evaluation. To establish the long-term clinical reliability and survival outcomes of BioHPP-based fixed implant restorations, high-quality randomized controlled trials with extended follow-up periods are imperative [40].

### Removable prostheses and bar-supported overdentures

BioHPP has garnered significant clinical interest in the field of removable prosthodontics, particularly in implant-retained overdenture systems utilizing bar frameworks. Its elastic modulus, ranging from approximately 3 to 4 GPa, closely resembles that of human cortical bone. This biomechanical compatibility facilitates more physiological stress distribution across supporting implants and surrounding bone, thereby reducing localized peri-implant stress and maintaining crestal bone levels [8, 41].

One of BioHPP’s key advantages is its excellent machinability through CAD/CAM workflows, which allows for the fabrication of anatomically precise, patient-specific bar structures that fulfill both functional and esthetic demands [23]. Clinical studies have shown that BioHPP bar frameworks provide comparable prosthetic retention to conventional metal designs. They also offer superior mucosal compatibility due to their low thermal conductivity and inert surface chemistry, contributing to enhanced soft tissue comfort [2].

Beyond its application in bar frameworks, BioHPP has also proven effective in the fabrication of esthetic clasp assemblies and occlusal rest components in removable partial dentures, particularly in clinical scenarios demanding enhanced esthetic integration and superior biocompatibility [5, 42, 43]. This versatility enhances BioHPP’s clinical utility in the rehabilitation of removable prosthetics. [44]. Saeedi et al. [44] demonstrated that CAD/CAM milling of BioHPP frameworks yields significantly greater dimensional accuracy than the conventional pressing technique, underscoring the precision advantages of digitally fabricated removable partial denture components. These findings underscore the critical role of selecting a manufacturing technique that aligns with the prosthesis’s structural and functional requirements. Furthermore, the material’s inherently low plaque affinity and high surface polish contribute to reduced bacterial colonization, thereby supporting peri-implant soft tissue health and promoting long-term clinical success and patient satisfaction [44].

### Abutments and provisional components

BioHPP has gained attention as a suitable material for provisional components, such as temporary abutments and crowns, because of its low bacterial affinity, excellent biocompatibility in transmucosal environments, and high dimensional stability [5, 16] These attributes are particularly advantageous during the healing and provisionalization phases, where maintaining peri-implant mucosal integrity and supporting optimal soft tissue architecture are critical for long-term esthetic and functional outcomes. According to Mohamed et al. [23], the seamless integration of high-performance polymer materials, such as BioHPP, into CAD/CAM workflows enables the rapid and precise fabrication of patient-specific prosthetic frameworks. This digital approach not only enhances clinical efficiency and consistency but also significantly reduces chairside time compared to conventional processing techniques. In addition to minimizing procedural time, the digital workflow enables precise control over the emergence profile. It facilitates superior esthetic integration, particularly in anterior regions with critical soft tissue contours.

Clinical investigations have shown that BioHPP-based provisional abutments offer dependable mechanical performance, promote favorable peri-implant soft tissue responses, and facilitate mucosal contour development during the early phases of healing [1, 45, 46].

In summary, BioHPP offers a biologically compatible, mechanically resilient, and digitally integrated framework for provisional components in implant prosthodontics, supporting both functional performance and esthetic predictability.

### Maxillofacial prosthetics

Beyond conventional intraoral prosthetic applications, BioHPP has demonstrated considerable potential in the field of maxillofacial rehabilitation, particularly in the restoration of extensive craniofacial defects. Its low density, superior biocompatibility, and inherent radiolucency confer distinct advantages in fabricating orbital prostheses, facial scaffolds, and obturators. These material attributes contribute to reduced prosthetic weight, improved patient comfort, and artifact-free postoperative imaging – an essential consideration for long-term clinical monitoring and outcome assessment. In a case report by Patil et al. [47], a two-piece obturator fabricated using BioHPP following maxillectomy, demonstrated successful functional integration alongside satisfactory esthetic rehabilitation. Similarly, numerous studies have supported the use of BioHPP in extraoral applications, emphasizing its lightweight structure, high dimensional accuracy through CAD/CAM workflows, and compatibility with diagnostic imaging modalities [47, 48].

### Esthetic applications

From an esthetic perspective, inherent opacity and limited translucency of BioHPP considerably restrict its use as a monolithic material in the anterior region, where high esthetic standards are required [49]. Porojan et al. [50] attributed these optical limitations to the semi-crystalline polymer matrix and embedded ceramic fillers, which induce light scattering rather than uniform transmission. Consequently, BioHPP is generally contraindicated for monolithic application in esthetically critical zones and veneering techniques are often adopted to overcome these shortcomings.

To address these limitations, bilayer restorative strategies involving nanohybrid composite resins or veneering ceramics have been widely employed [19, 50]. These bilayer approaches enhance translucency and chromatic integration, thereby improving the overall esthetic outcome. However, their long-term success is highly dependent on the integrity of the adhesive interface and the surface conditioning. Recommended protocols include airborne-particle abrasion, plasma surface modification, and the application of MMA-based primers to promote durable adhesion [29].

Moreover, when BioHPP is used as a framework material, its favorable surface characteristics, excellent biocompatibility, and minimal plaque accumulation facilitate integration with peri-implant soft tissues. This contributes to mucosal seal stability and enables a natural-looking emergence profile, which is particularly critical in the esthetic zone [1]. A randomized controlled clinical trial by El-Shimy et al. [51] compared BioHPP-based single-unit restorations (veneered with composite) to porcelain-veneered zirconia crowns. After a 1-year follow-up, both groups demonstrated comparable and favorable outcomes in terms of color stability, mechanical integrity, and soft tissue response.

## Discussion

### Comparison with conventional framework materials

The selection of an appropriate framework material in prosthetic dentistry plays a pivotal role in determining the long-term clinical performance of restorations. BioHPP – a high-performance polymer derived from PEEK and reinforced with 20% ceramic fillers – offers a distinctive blend of biomechanical compatibility, biocompatibility, and seamless integration into digital workflows. Nevertheless, as with all biomaterials, certain limitations remain when compared to well-established framework materials such as titanium, cobalt–chromium (Co–Cr) alloys, and zirconia [1, 8].

Mechanically, BioHPP exhibits an elastic modulus of approximately 3–4 GPa, substantially lower than that of titanium (~110 GPa), Co–Cr alloys (~200 GPa), and zirconia (~200 GPa). This bone-mimetic modulus closely approximates that of human cortical bone (~14 GPa), enabling more physiological load transfer and mitigating stress shielding effects [3, 14]. However, its relatively lower surface hardness and fracture toughness may make it less suitable for restorations in high-load bearing areas, such as distal cantilevers and molar regions [37].

BioHPP demonstrates superior tissue compatibility from a biological perspective compared to metallic alternatives. Its chemical inertness, absence of galvanic corrosion, and lack of ion release prevent mucosal pigmentation and hypersensitivity reactions, which contribute to a stable peri-implant environment [1]. Studies comparing BioHPP and Co–Cr bar frameworks in implant-supported overdentures indicate significantly reduced mucosal inflammation and vertical bone loss with BioHPP [2, 36].

Although BioHPP is opaque and requires veneering for anterior applications, its radiolucency facilitates artifact-free radiographic assessment – an advantage not shared by zirconia or metal-based restorations [52]. This characteristic is beneficial for long-term monitoring of peri-implant bone levels using CBCT or periapical radiographs.

Regarding manufacturing, BioHPP is entirely compatible with CAD/CAM technologies, removing the necessity for casting or sintering and enabling the creation of precise, lightweight, and reproducible prostheses [6, 12]. However, its low surface energy and hydrophobicity present a bonding challenge, requiring specific surface conditioning protocols – such as plasma treatment or sulfuric acid etching – followed by the use of PAEK-compatible primers and adhesives based on functional monomers [53, 54].

In summary, BioHPP represents a promising alternative to conventional framework materials by combining favorable biomechanical properties, enhanced soft tissue biocompatibility, and seamless integration into fully digital prosthodontic workflows. Nevertheless, achieving optimal clinical performance and long-term prosthesis durability requires careful case selection, reliable bonding protocols, and reinforcement in regions exposed to high functional loads.

## Limitations and future perspectives

BioHPP has generated significant interest in prosthetic dentistry due to its beneficial biomechanical properties, biocompatibility, and alignment with digital manufacturing workflows. However, several limitations have been repeatedly noted in the existing literature, which must be critically evaluated for broader clinical adoption.

The material’s low surface energy and intrinsic hydrophobicity compromise its adhesive capacity, hindering reliable chemical bonding with veneering resins and luting agents. Although surface treatments such as airborne particle abrasion, plasma activation, and sulfuric acid etching have been developed to improve adhesion, bond durability remains a concern. Studies have shown a significant reduction in bond strength following thermal cycling and mechanical aging, indicating potential limitations in long-term clinical reliability [9, 55].

Mechanically, while BioHPP’s low elastic modulus provides a stress-dampening advantage by mimicking the viscoelastic behavior of cortical bone, its relatively low surface hardness and limited fracture toughness restrict its use in high-load posterior restorations and monolithic applications. The material’s inherent opacity and susceptibility to color instability pose challenges in anterior restorations, particularly when high translucency is required.

Furthermore, although extensive in vitro research has clarified BioHPP’s structural and adhesive properties, long-term, randomized, controlled clinical trials are still lacking [1, 56]. This evidence gap limits the ability to evaluate its performance in real-world clinical conditions. Additionally, there is no consensus on optimal bonding protocols, and comparative analyses of existing surface treatment strategies are insufficiently documented [1].

Future research should prioritize generating high-quality clinical evidence through prospective, multicenter trials that evaluate the performance of BioHPP across diverse prosthetic indications. In parallel, advancements in nanofiller incorporation, hybrid polymer development, and innovative surface functionalization techniques hold promise for enhancing the material’s mechanical strength and adhesive potential. Equally essential is the promotion of interdisciplinary collaboration aimed at improving esthetic outcomes and ensuring long-term interfacial stability in complex clinical scenarios.

In conclusion, BioHPP represents a promising framework material for modern prosthetic dentistry. However, its widespread clinical adoption will rely on ongoing innovation in materials science, rigorous clinical validation, and the creation of standardized adhesive protocols tailored to its unique surface chemistry. Nevertheless, long-term comparative clinical data remain limited, emphasizing the need for continued investigation of BioHPP’s mechanical and adhesive reliability under functional conditions.

## Conclusions

Owing to its bone-mimetic elastic modulus, chemical inertness, and low plaque affinity, BioHPP has garnered increasing attention as a high-performance alternative to conventional framework materials in prosthetic dentistry. An expanding body of evidence supports its effective use in both fixed and removable prosthetic applications, particularly in optimizing stress distribution, preserving peri-implant tissue health, and enhancing patient comfort.

Nonetheless, inherent limitations in surface bonding and translucency necessitate the use of specialized surface treatments and careful case selection, especially in esthetically demanding or high-load-bearing restorations. Moreover, well-designed long-term clinical trials are essential to confirm BioHPP’s suitability as a mainstream prosthetic material across diverse indications. Concurrently, interdisciplinary research aimed at improving adhesive performance and esthetic integration will be crucial for fully realizing its clinical potential.

## Data Availability

Data sharing does not apply to this article as no new data were created or analyzed in this study.

## References

[1] Reda R, Zanza A, Galli M, De Biase A, Testarelli L, Di Nardo D. Applications and clinical behavior of BioHPP in prosthetic dentistry: a short review. J Compos Sci. 2022;6(3):90. 10.3390/jcs6030090

[2] Moussa SG, El Afandy HM. Patient satisfaction and soft tissue changes around BioHPP bar compared to cobalt-chromium bar retaining mandibular implant-overdentures: a randomized clinical trial. Al-Azhar J Dent Sci. 2023;26(3):381–90. 10.21608/ajdsm.2023.213530.1434

[3] Bathala L, Majeti V, Rachuri N, Singh N, Gedela S. The role of polyether-ether-ketone (PEEK) in dentistry – a review. J Med Life. 2019;12(1):5. 10.25122/jml-2019-0003 31123518 PMC6527406

[4] Georgiev J, Vlahova A, Kissov H, Aleksandrov S, Kazakova R. Possible application of BioHPP in prosthetic dentistry: a literature review. J IMAB. 2018;24(1):1896–8. 10.5272/jimab.2018241.1896

[5] Zoidis P, Papathanasiou I, Polyzois G. The use of a modified poly-ether-ether-ketone (PEEK) as an alternative framework material for removable dental prostheses: a clinical report. J Prosthodont. 2016;25(7):580–4. 10.1111/jopr.12325 26216668

[6] Wei X, Wang M, Pan Y, Lin H, Jiang L, Wang Y, et al. Influence of fabrication method on the biological properties of modified PEEK. Dent Mater J. 2023;42(1):72–8. 10.4012/dmj.2022-115 36351597

[7] Gama LT, Bezerra AP, Schimmel M, Garcia RCMR, de Luca Canto G, Gonçalves TMSV. Clinical performance of polymer frameworks in dental prostheses: a systematic review. J Prosthet Dent. 2024;131(4):579–90. 10.1016/j.prosdent.2022.03.002 35422333

[8] Guo L, Smeets R, Kluwe L, Hartjen P, Barbeck M, Cacaci C, et al. Cytocompatibility of titanium, zirconia and modified PEEK after surface treatment using UV light or non-thermal plasma. Int J Mol Sci. 2019;20(22):5596. 10.3390/ijms20225596 31717459 PMC6888564

[9] Arslan E, Caglar I. Enhancing PEEK bond strength: the impact of chemical and mechanical surface modifications on surface characteristics and phase transformation. BMC Oral Health. 2025;25(1):511. 10.1186/s12903-025-05933-3 40211271 PMC11983897

[10] Wiesli MG, Özcan M. High-performance polymers and their potential application as medical and oral implant materials: a review. Implant Dent. 2015;24(4):448–57. 10.1097/ID.0000000000000285 26035377

[11] Liu Y, Fang M, Zhao R, Liu H, Li K, Tian M, et al. Clinical applications of polyether-ether-ketone in removable dental prostheses: accuracy, characteristics, and performance. Polymers (Basel). 2022;14(21):4615. 10.3390/polym14214615 36365609 PMC9654455

[12] Erdaş G, Özdemir H. The use of PEEK and PEKK in prosthodontics. Curr Res Dent Sci. 2023; (early online).

[13] Amjadi M, Safari MR, Dini Torkamani R, Kheiri Manjili M, Jamshidi D, Sobhan F, et al. Comparative assessment of post-fatigue resistance of mandibular first molars restored with polyether-ether-ketone and lithium disilicate endocrowns. Sci World J. 2024;2024:8648372. 10.1155/tswj/8648372 PMC11617050 39633959

[14] Kandeel ZM, Abdelhamed AM, Neena AF. Strain developed around dental implants loaded with two CAD-CAM reinforced polymeric superstructure materials: an in vitro comparative study. Alex Dent J. 2023;48(2):124–31. 10.21608/adjalexu.2022.127546.1262

[15] Elshamy AH, Abdel Kader SH, Nassif MS. The effect of superstructure material on stress distribution in bone around implants supporting fixed partial denture: an in vitro study. Alex Dent J. 2022;47(3):116–24. 10.21608/adjalexu.2021.71459.1182

[16] Andrikopoulou E, Zoidis P, Artopoulou II, Doukoudakis A. Modified PEEK resin-bonded fixed dental prosthesis for a young cleft lip and palate patient. J Esthet Restor Dent. 2016;28(4):201–7. 10.1111/jerd.12221 27273727

[17] Ahmed GA, El-Etreby A, El-Fattah GA. Comparison of shear bond strength and color reproduction of two different high-performance polymers veneered with two different thicknesses of resin composite: an in vitro study. Braz Dent Sci. 2023;26(4) :e3959. 10.4322/bds.2023.e3959.

[18] Younes AA, Korsel AM, El Tokhey HM, Ali KM, Kamel MS. The effect of different implant abutment materials on the stress distribution to the bone–implant contact. Egypt Dent J. 2020;66(2):1289–94. 10.21608/edj.2020.25438.1062

[19] Beleidy M, Ziada A. Marginal accuracy and fracture resistance of posterior crowns fabricated from CAD/CAM PEEK cores veneered with HIPC or nanohybrid conventional composite. Egypt Dent J. 2020;66(4):2541–52.

[20] Elsebai DII, Eid HI, Osama AM, Mohamed HT. Effect of CAD/CAM-constructed BioHPP versus zirconia frameworks reinforced maxillary complete denture on fracture resistance: an in vitro study. Open Access Maced J Med Sci. 2023;11(D):55–60. 10.3889/oamjms.2023.11546

[21] Al-Asad HM, El Afandy MH, Mohamed HT, Mohamed MH. Hybrid prosthesis versus overdenture: effect of BioHPP prosthetic design rehabilitating edentulous mandible. Int J Dent. 2023;2023:4108679. 10.1155/2023/4108679 37426766 PMC10325880

[22] Hanno K. Full-mouth implant rehabilitation using fixed-detachable BioHPP frameworks in the presence of one-piece implants: a clinical report. J Dent Health Oral Res. 2022;3(2):1–10.

[23] Mohamed MN, Mohamed GF, Hassan NO. Comparative evaluation of physical properties of conventionally constructed versus CAD/CAM-milled frameworks for Kennedy Class I partial dentures. Egypt Dent J. 2019;65(4):3663–70. 10.21608/edj.2019.75998

[24] Lo Giudice R, Puleio F, Matarese M, Nicita F, Pantè GG, Previti C, et al. BioHPP and soft tissue: confocal laser scanning evaluation of junctional connective tissue. Clin Oral Implants Res. 2019;30(Suppl 18):283–4. 10.1111/clr.239_13509

[25] Discepoli N, Mirra R, De Rubertis I, Ricciardi R, La Francesca N, Bellan C. Histopathologic assessment of soft-tissue healing around polyether-ether-ketone and titanium abutments: a randomized controlled human study. J Osseointegr. 2025;17(1):40–7. 10.1155/ijod/7500003

[26] Montaser A. Evaluation of marginal fit and fracture resistance of zirconia implant abutment supporting two types of metal-free CAD/CAM restorations. Al-Azhar J Dent. 2022;9(2):14.

[27] Almuhayya S, Alshahrani R, Alsania R, Albassam A, Alnemari H, Babaier R. Biofilm formation on three high-performance polymeric CAD/CAM composites: an in vitro study. Polymers (Basel). 2025;17(5):676. 10.3390/polym17050676 40076168 PMC11902371

[28] Lee E-H, Lee S-W, Seo Y, Deng Y-H, Lim Y-J, Kwon H-B, et al. Manganese-oxide nanozyme-doped diatom for safe and efficient treatment of peri-implantitis. ACS Appl Mater Interfaces. 2022;14(24):27634–50. 35638645 10.1021/acsami.2c05166 PMC11445715

[29] Luo C, Liu Y, Peng B, Chen M, Liu Z, Li Z, et al. PEEK for oral applications: recent advances in mechanical and adhesive properties. Polymers (Basel). 2023;15(2):386. 10.3390/polym15020386 36679266 PMC9864167

[30] Sarfaraz H, Rasheed MN, Shetty SK, Prabhu UM, Fernandes K, Mohandas S. Comparison of the bond strength of composite resin to zirconia and to polyether-ether-ketone: an in vitro study. J Pharm Bioallied Sci. 2020;12(Suppl 1):S504–9. 10.4103/jpbs.JPBS_147_20 33149512 PMC7595458

[31] Erjavec AK, Črešnar KP, Švab I, Vuherer T, Žigon M, Brunčko M. Determination of shear bond strength between PEEK composites and veneering composites for the production of dental restorations. Materials (Basel). 2023;16(9):3286. 37176168 10.3390/ma16093286 PMC10178894

[32] Adeeb Gabra EN, Sadek HMA, Hamdy AM, Wahsh MM. Effect of surface treatment and resin cement type on the bond strength of polyether-ether-ketone to lithium-disilicate ceramic. BMC Oral Health. 2024;24(1):513. 10.1186/s12903-024-04269-8 38698366 PMC11064278

[33] El-Wassefy NA. Shear bond strength of two veneering composite resins to a modified polyether-ether-ketone material: influence of surface pretreatments and thermocycling. Egypt Dent J. 2019;65(3):2821–30. 10.21608/edj.2019.72677

[34] Gonçalves TMSV, Reginaldo I, Baroudi K, Wanghon ZML, Santos Diamantino P, Gimenez MG, et al. Effect of adhesive system on bond strength of PEEK and PEKK. J Compos Sci. 2025;9(4):165.

[35] Yousry MA, Hussein SA, Al Abbassy FH. Evaluation of shear bond strength of high-performance polymers to its resin veneering and to dentIn: an in vitro study. Alex Dent J. 2018;43(2):62–8. 10.21608/adjalexu.2018.57626

[36] Alnafaiy SM, Labban N, Albaijan R, AlKahtani RN, Al-Aali KA, Abozaed HW, et al. Evaluation of shear bond strength and failure modes of lithium-disilicate ceramic veneering material to different high-performance polymers. Polymers (Basel). 2025;17(5):554. 10.3390/polym17050554 40076048 PMC11901903

[37] Kowalski R, Frąckiewicz W, Kwiatkowska M, Światłowska-Bajzert M, Sobolewska E. Comparison of performance parameters of BioHPP and Biocetal used in the production of prosthetic restorations in dentistry: part I, mechanical tests. Materials (Basel). 2025;18(3):561. 10.3390/ma18030561 39942228 PMC11818239

[38] Aboelnagga MM. Comparative stress analysis of BioHPP and PEKK CAD/CAM frameworks in mandibular All-on-Four fixed-detachable prosthesis on its supporting implants. Ain Shams Dent J. 2023;32(4):6–18.

[39] Shash YH, El-Wakad MT, Eldosoky MA, Dohiem MM. Evaluation of stress and strain on mandible caused using ‘All-on-Four’ system from PEEK in hybrid prosthesis: finite-element analysis. Odontology. 2023;111(3):618–29. 10.1007/s10266-022-00771-z 36436151 PMC10238352

[40] Jin H-Y, Teng M-H, Wang Z-J, Li X, Liang J-Y, Wang W-X, et al. Comparative evaluation of BioHPP and titanium as frameworks veneered with composite resin for implant-supported fixed dental prostheses. J Prosthet Dent. 2019;122(4):383–8. 10.1016/j.prosdent.2019.03.003 30982624

[41] Nabhan MS. Effect of different fixed-detachable implant-supported prosthesis materials on stresses induced on supporting structures. Egypt Dent J. 2019;65(1):445–52. 10.21608/edj.2019.72720

[42] Güneş F, Kocacıklı M, Korkmaz T. Dental implantolojide polieter-eter-keton (PEEK): geleneksel derleme. Selcuk Dent J. 2023;10(3):611–7. 10.15311/selcukdentj.1238899

[43] Sen S, Mondal S, Mukhopadhyay S, Maji S, Das S. Esthetic clasp material: a review. J Orofac Rehabil. 2022;2(2):28–36.

[44] Saeedi E, Sayed TMA, Thabet YG, Mohamed SL, Sabet ME. Evaluation of the accuracy and adaptation of BioHPP removable partial denture frameworks constructed by milling vs the pressing technique. Int J Prosthodont. 2022;35(5):647–52. 10.11607/ijp.7822 36511791

[45] Hanna EG, Amine S, Prasad B, Younes K. Exploring polyether-ether-ketone in dental implants and abutments: a focus on biomechanics and finite-element methods. Rev Adv Mater Sci. 2024;63(1):20240031. 10.1515/rams-2024-0031

[46] Pascalis FD. Soft-tissue integration with a hybrid abutment using the ‘one abutment–one time’ therapeutic protocol: case series. Quintessence Int. 2022;53(7):703–8. 10.3290/j.qi.b3082565 35723484

[47] Patil AA, Kalsekar B, Kasat SJ, Patil SS. Fabrication of maxillary obturator using a combination of PEEK, acrylic resin, and silicone: a case report. Int J Prosthodont Restor Dent. 2022;12(1):46–50. 10.5005/jp-journals-10019-1357

[48] El-Hussein IG. Impact of milled PEEK versus conventional chrome-cobalt framework on retention of definitive obturators for unilateral total maxillectomy patients. Al-Azhar J Dent. 2024;10(4):22. 10.58675/2974-4164.1607

[49] Gouda A, Sherif A, Wahba M, Morsi T. Effect of veneering material type and thickness ratio on flexural strength of bi-layered PEEK restorations before and after thermal cycling. Clin Oral Investig. 2023;27(6):2629–39. 10.1007/s00784-022-04829-8 PMC10264476 36602589

[50] Porojan L, Toma FR, Bîrdeanu MI, Vasiliu RD, Uțu I-D, Matichescu A. Surface characteristics and color stability of dental PEEK related to water saturation and thermal cycling. Polymers (Basel). 2022;14(11):2144. 10.3390/polym14112144 35683817 PMC9183185

[51] El-Shimy KRAE, Zaki A. One-year clinical evaluation of pressed BioHPP PEEK-based versus zirconia-based single crowns: randomized controlled clinical trial. NeuroQuantology. 2022;20(17):901–14.

[52] Rajamani VK, Reyal SS, Gowda EM, Shashidhar MP. Comparative prospective clinical evaluation of CAD/CAM-milled BioHPP PEEK inlays and zirconia inlays. J Indian Prosthodont Soc. 2021;21(3):240–8. 10.4103/jips.jips_57_21 34380810 PMC8425372

[53] Fareed AM, Elsheehy OM, Nabil O. Efficacy of two surface treatment methods of PEEK on shear bond strength to veneering composite: an in vitro study. Adv Dent J. 2023;5(4):734–41. 10.21608/adjc.2023.228885.1381

[54] Gama LT, Duque TM, Özcan M, Philippi AG, Mezzomo LAM, Gonçalves TMSV. Adhesion to high-performance polymers applied in dentistry: a systematic review. Dent Mater. 2020;36(4):e93–108. 10.1016/j.dental.2020.01.002 32035670

[55] Robaian A, Alshehri AM, Alqhtani NR, Almudahi A, Alanazi KK, Abuelqomsan MA, et al. Effect of surface treatments and thermal aging on bond strength between veneering resin and CAD/CAM provisional materials. Polymers (Basel). 2025;17(5):563. 10.3390/polym17050563 40076056 PMC11902269

[56] Speroni S, Antonelli L, Coccoluto L, Giuffrè M, Sarnelli F, Tura T, et al. The effectiveness and predictability of BioHPP superstructures in Toronto–Brånemark implant-prosthetic rehabilitations: a case report. Prosthesis. 2025;7(1):10. 10.3390/prosthesis7010010

